# Cardio-respiratory deep learning model to predict hospital admission from emergency department electrocardiogram images and respiration videos: a prospective study

**DOI:** 10.1186/s13040-026-00583-9

**Published:** 2026-07-08

**Authors:** Guang-Yuan Chen, Shao-Chi Lu, Wen-Hsiang Cheng, Jun-Wan Gao, Jen-Tang Sun, Chien-Hua Huang, Chu-Lin Tsai, Li-Chen Fu

**Affiliations:** 1 Graduate Institute of Networking and Multimedia, National Taiwan University, Taipei, Taiwan; 2 Department of Emergency Medicine, National Taiwan University Hospital, National Taiwan University College of Medicine, Taipei, Taiwan; 3 Department of Emergency Medicine, Far Eastern Memorial Hospital, New Taipei City, Taiwan; 4 Department of Computer Science and Information Engineering, National Taiwan University, CSIE Der Tian Hall, No. 1, Section 4, Roosevelt Road, Taipei, 106319 Taiwan

**Keywords:** Admission, Electrocardiography, Respiration, Deep Learning, Computer Vision

## Abstract

**Background:**

Early prediction of hospital admission at the emergency department (ED) triage can improve patient flow and resource allocation. Most existing models rely solely on structured data. Incorporating multimodal physiologic information, such as cardiac and respiratory signals, may better capture subtle clinical signs. This study aimed to develop a deep learning fusion model that integrates electrocardiogram (ECG) images and respiration videos to predict hospital admission at ED triage.

**Methods:**

We prospectively collected ECG images and respiration videos from adult ED patients in a tertiary medical center. The proposed model consisted of modality-specific branches: an ECG branch using transfer learning, a respiration-signal-estimation pipeline, and a spatial attention module to enhance cardiopulmonary feature extraction. Class-balanced loss was applied to address dataset imbalance. Model performance was compared with the baseline Taiwan Triage Acuity Score (TTAS) and single-modality variants. Attention heatmaps using score-class activation mapping (score-CAM) were analyzed for interpretability.

**Results:**

The study included 118 patients, with a 27% admission rate. The ECG-respiration fusion model achieved an accuracy of 0.814, a recall of 0.749, a precision of 0.635, an F1-score of 0.680, an area under the receiver operating characteristic curve (AUROC) of 0.863, and an area under the precision-recall curve (AUPRC) of 0.675, outperforming both TTAS and single-modality models. Ablation analyses confirmed the additive value of spatial attention and class-balanced loss. Score-CAM visualizations indicated the model focused on physiologically relevant features, such as respiratory waveform inflection points.

**Conclusions:**

A multimodal deep learning approach combining ECG and respiration video signals can predict hospital admission at ED triage. Integrating such models into triage systems may enhance earlier risk identification and resource prioritization.

**Clinical trial number:**

Not applicable.

**Supplementary Information:**

The online version contains supplementary material available at https://doi.org/10.1186/s13040-026-00583-9.

## Introduction

Emergency department (ED) overcrowding has emerged as a global public health crisis [1], compromising timely patient care and straining healthcare resources. Overcrowding reduces ED capacity, increases waiting times and ambulance diversions, and elevates the risk of adverse outcomes, including higher inpatient mortality and longer lengths of stay [2, 3]. Existing triage systems, such as the Emergency Severity Index (ESI) [4] and the Taiwan Triage and Acuity Scale (TTAS) [5], rely largely on structured data (e.g., demographics, vital signs), but often fail to predict patient disposition accurately, leading to inefficiencies in allocating limited resources in the ED.

In recent years, computer vision has achieved significant milestones in medical applications [6], enabling video-based AI systems to perform tasks across various clinical scenarios [7–10], demonstrating the feasibility of leveraging AI for continuous and non-invasive patient evaluation. However, real-time video analysis for ED triage, which aims to emulate clinicians’ rapid “clinical gestalt” [11, 12] based on patient appearance, remains largely unexplored. Clinicians can achieve approximately 80% accuracy in disposition decisions from brief (~ 30 s) visual observations [13], yet human judgment in busy ED environments is prone to variability and limited by staffing constraints. To our knowledge, there has been only one study using computer vision AI with short patient video clips to predict hospitalization from the ED [14]. Harnessing deep learning to extract richer clinical cues from both respiratory videos and ECG imagery holds promise for more accurate admission-risk prediction.

Accurate predictions of hospital admissions enable emergency departments to allocate beds, staff, and monitoring equipment more effectively, reducing bottlenecks and wait times. By flagging patients with high admission risk early (e.g., at triage), clinicians can prioritize critical cases and expedite necessary interventions. On the other hand, better forecasts prevent unnecessary admissions for low-risk patients, conserving scarce inpatient resources and reducing crowding downstream. In both ways, robust admission-prediction models directly translate into smoother ED workflows. During triage, a patient’s electrocardiogram (ECG) (if available) and respiratory pattern provide rich information for risk stratification. ECG features, such as waveform morphology, carry early clues of cardiac compromise [15], while respiration dynamics reflect both pulmonary function and possible compensatory reactions due to other stress. When analyzed separately, each modality offers only a partial view of a patient’s physiological state. A model that leverages both data streams simultaneously can discover synergistic markers, subtle but predictive interactions between cardiac electrical activity and breathing patterns that would otherwise remain hidden. Unlike traditional scoring systems or single modality approaches, combining ECG images with real-time respiratory videos represents a new frontier in non‐invasive cardiopulmonary evaluation.

In this study, we aimed to develop a cardio-respiratory model that can process synchronized signals, mirroring clinicians’ visual “clinical gestalt” but with greater consistency and objectivity. This multimodal strategy not only accelerates risk assessment, since both inputs are captured during triage, but also enables more granular feature learning, such as cross-modal attention between cardiac and respiratory indicators.

## Methods

### Study design, setting, and population

We prospectively collected data between February and December 2024 in the emergency department (ED) of National Taiwan University Hospital (NTUH), a tertiary academic medical center in Taiwan with approximately 2,400 beds and an annual ED volume of about 100,000 visits. Trained research staff consecutively enrolled eligible patients following a standardized protocol. Inclusion criteria were age ≥ 18 years and the ability to provide informed consent. Patients were excluded if they required immediate resuscitation, were unconscious without a legal representative, had severe psychiatric disorders impeding communication, or had infectious diseases necessitating isolation. The study was approved by the NTUH Institutional Review Board (reference number: 202308069RINA), and written informed consent was obtained from all participants. In total, 118 patients were enrolled in the primary cohort. The participant flow chart is shown in the Online Supplemental Material. With the securement of another round of funding, we have continued the enrollment since January 27, 2026. As of March 10, 20 patients have been available for independent validation. Among the 20 patients, 10 had an ECG at triage.

During triage under ambient lighting, a 30-second video of each participant’s lower face (covered by a face mask), neck, and clothed chest wall was recorded using an iPhone 13 (Apple Inc., CA, USA) mounted on a stand. The recordings were primarily front-facing, though the camera angle for some seated patients could vary within approximately ± 50 degrees from the frontal orientation. Patients with limited mobility were filmed in wheelchairs, while those in more severe condition were recorded lying on beds. Respiratory rate labels were annotated by board-certified emergency physicians who reviewed chest movements through repeated video playback. These physician-annotated respiratory rates served as a reference standard for verifying the breath rates derived from the extracted respiratory waveform (see Methods, Respiratory signal estimation).

According to ED policy, patients presenting with symptoms suggestive of acute coronary syndrome, arrhythmia, syncope, or other serious conditions identified by triage nurses routinely underwent a 12-lead electrocardiogram (ECG) at triage, with resulting ECG reports stored in portable document format (PDF).

### System overview

**Fig. 1:**
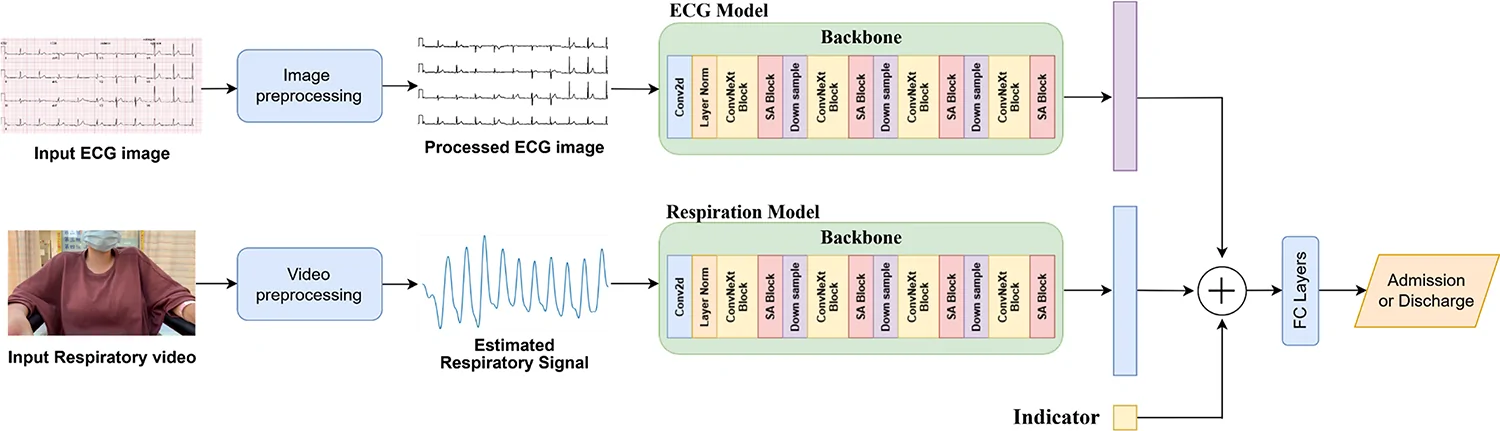
The overview of the proposed model. SA indicates the Spatial Attention Module. The indicator specifies whether a patient has ECG data available

We propose a multimodal deep learning model that integrates respiratory videos and ECG images to predict hospital admission in a real‑world ED setting without using structured triage data. The overview of the designed model is shown in Fig. 1. At the input stage, we input two data streams collected from the ED of NTUH: ECG images extracted from standard 12‑lead ECG PDF reports and patient respiration videos recorded at triage. The ECG images are first processed by an image‑preprocessing pipeline, while the videos are passed through a respiratory‑signal‑estimation pipeline to produce denoised waveform representations. Each modality is then fed into its own ConvNeXt‑T [16] backbone, augmented with a Spatial Attention Module to enhance feature extraction. To compensate for limited ECG samples, the ECG branch is pretrained on an external ECG dataset. In the fusion stage, the two feature vectors are concatenated with a binary indicator that indicates whether ECG data are present, and passed through a series of fully connected layers to predict hospital admission. Finally, to mitigate class imbalance in our training set, we employ a Class‑Balanced loss [17]. Further implementation details are provided in the subsequent sections.

### Data preprocessing

#### Respiration video preprocessing pipeline overview

**Fig. 2:**
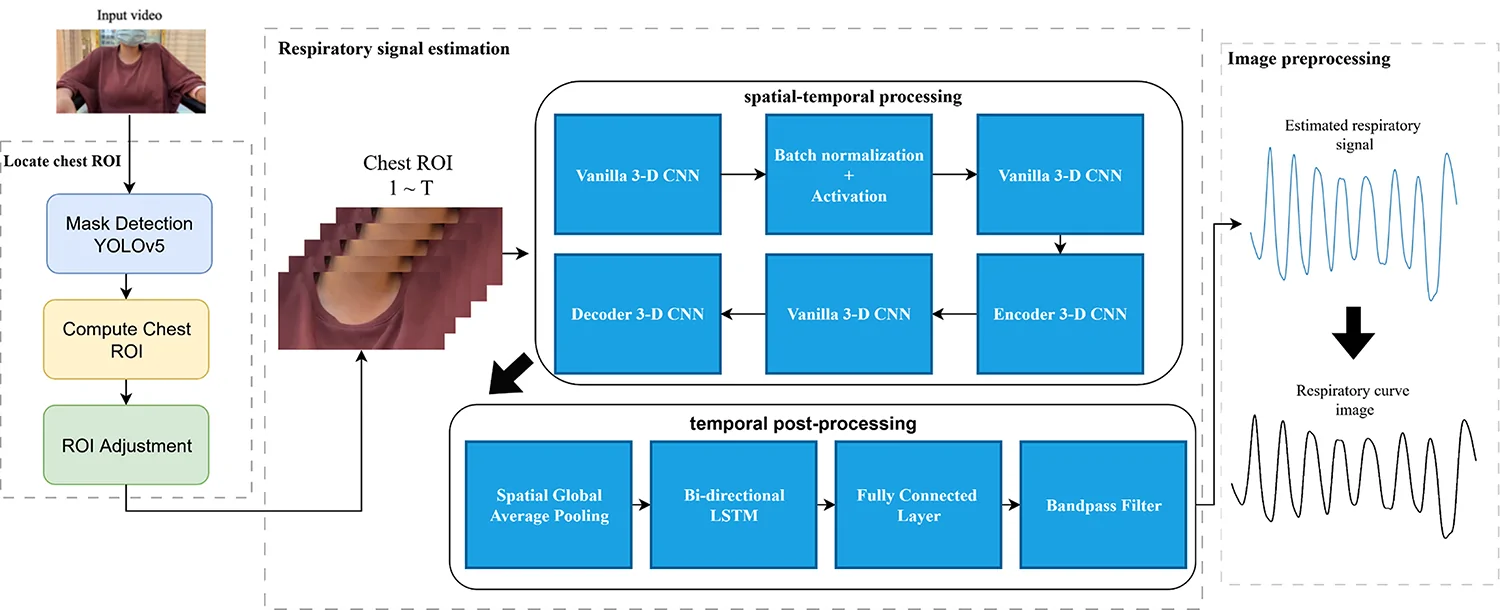
The overview of our respiration video preprocessing pipeline

The overview of the designed pipeline is shown in Fig. 2. Patient respiration videos recorded in NTUH were first decoded into individual frames according to the original frame rate, and each frame was then processed through three sequential stages. First, we used deep learning models to automatically localize the chest Region of Interest (ROI) in each frame, isolating the area relevant to respiratory motion. Second, following the method of Mozafari et al. [18], the cropped chest-ROI image sequence was fed into a 3D convolutional neural network (CNN), a bi‐directional long short‑term memory stage, and a fully connected layer to estimate the raw respiratory waveform, which was subsequently denoised using a band‑pass filter. Finally, the cleaned respiratory signal underwent simple processing and was converted into a standardized respiratory curve image suitable for model input.

#### Chest ROI selection

To preserve patient privacy, collected videos showed only the masked lower face. For each frame, we first detected the mask region and derived the chest ROI based on its position. A YOLOv5 [19] detector fine-tuned on the Face-Mask dataset (≈ 1,000 annotated images) achieved a recall of 0.90 and a mean average precision (mAP) of 0.95 for mask localization. During inference on ED videos, which often contained multiple people or cluttered backgrounds, we retained the bounding box with the largest area as the participant’s mask and placed a rectangular chest ROI directly below it.

#### Respiratory signal estimation

**Fig. 3:**
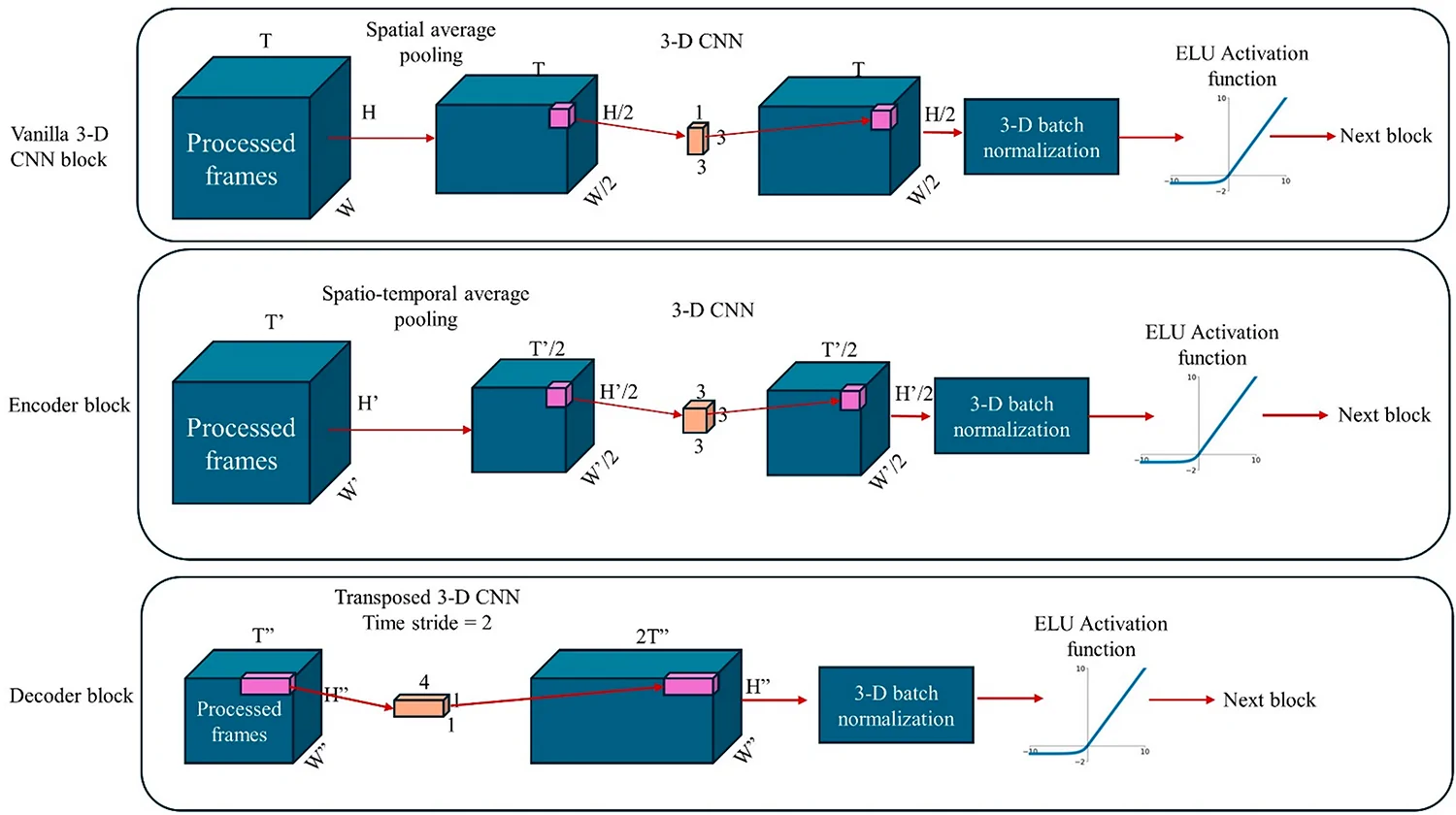
Illustration of vanilla, encoder, and decoder 3D CNN blocks [18]

To extract the respiratory curve from the chest ROI, we adopted the spatio-temporal deep learning pipeline of Mozafari et al. [18], which builds on the temporal encoder–decoder 3D CNN architecture introduced by Lea et al. [20]. Chest‑ROI frame sequences were first passed through temporal encoder blocks, where average pooling over both spatial and temporal dimensions reduced width, height, and sequence length. Decoder blocks then used transpose 3D convolutions with strides greater than one to restore the original temporal resolution. Between these stages, vanilla 3D CNN blocks refined spatial features without changing the time dimension. Figure 3 illustrates each block type.

To capture long-term dependencies beyond the capacity of 3D CNNs alone, we appended a many-to-many bi-directional LSTM layer that processed a 10-second sliding window of encoder outputs in both forward and backward directions [18]. Its outputs were fed into a fully connected layer to produce a raw respiratory waveform, which was then band-pass filtered in the range of 3 to 60 breaths per minute to remove noise outside physiological frequencies.

The estimator was trained against reference respiratory waveforms derived from the chest-ROI image sequences using the established non-contact RGB respiration-measurement method of Massaroni et al. [21], with a Savitzky–Golay filter applied to suppress residual noise. To verify that the extracted signal captured genuine respiratory dynamics, breath rates derived from the bandpass-filtered waveform via peak detection were compared against physician-annotated respiratory rates recorded at patient enrollment, yielding a Pearson correlation coefficient of 0.73.

#### Respiratory curve image preprocessing

**Fig. 4:**
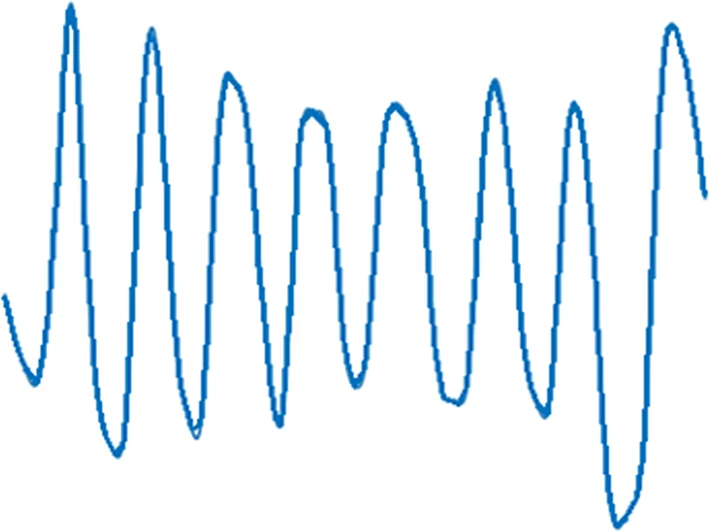
Raw respiratory curve image

**Fig. 5:**
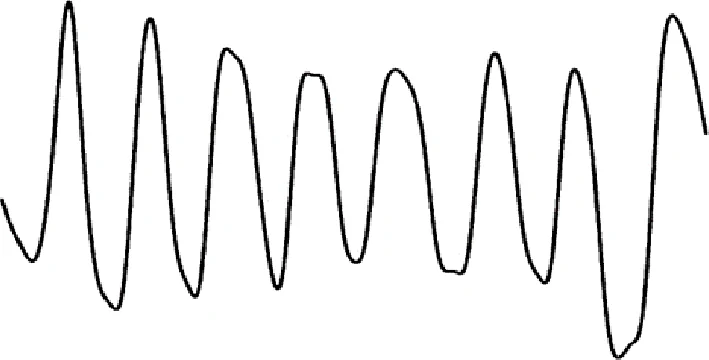
Preprocessed respiratory curve image

After noise reduction, the resulting respiratory signal was transformed into an image representation, as illustrated in Fig. 4. This image subsequently underwent a series of preprocessing steps, including resizing, morphological opening, and binarization. These operations were intended to enhance the clarity and uniformity of the curve image, thereby improving its suitability for input into the model. The final preprocessed result is shown in Fig. 5.

#### ECG image preprocessing

**Fig. 6:**
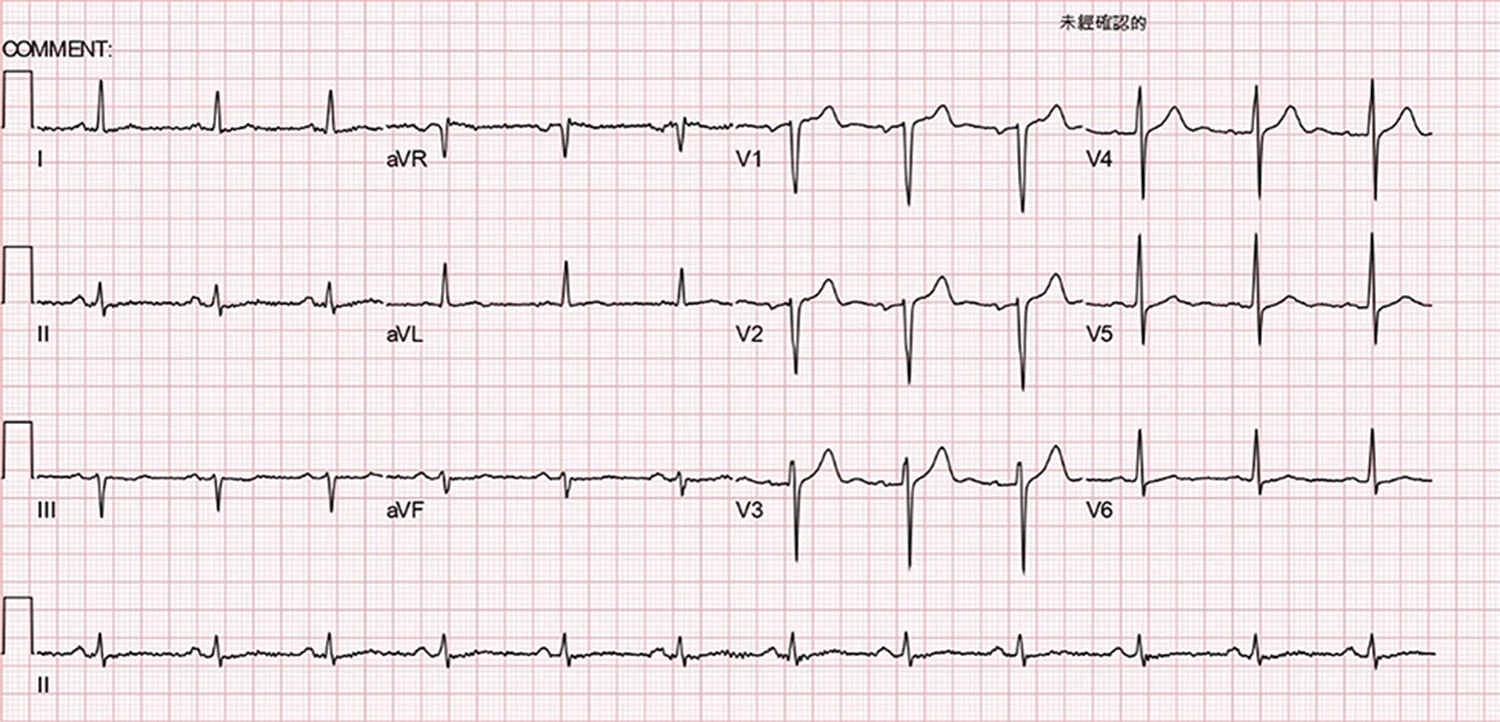
The original image extracted from the ECG report

As shown in Fig. 6, raw ECG images often contain auxiliary visual elements such as red grid lines and lead annotations, which are helpful for human interpretation but may introduce noise to learning-based models. Therefore, we applied the dedicated preprocessing pipeline [22] to remove these extraneous components.

**Fig. 7:**
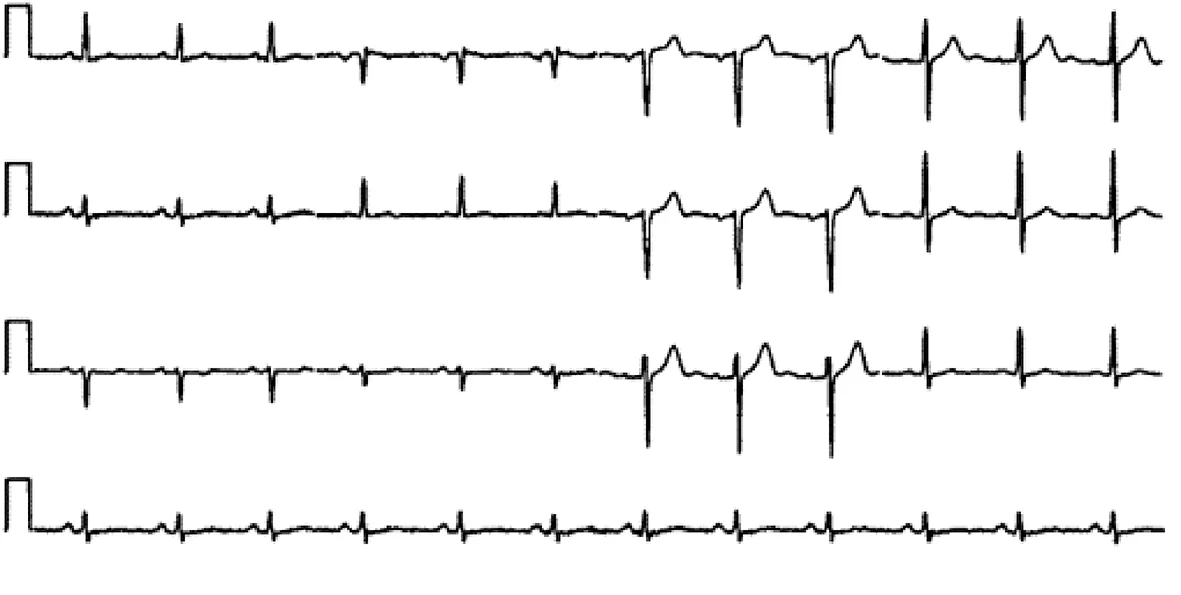
The ECG image after preprocessing

The red channel was first thresholded to remove red grid lines and binarize the image, effectively eliminating background grids while preserving waveform structure. Contour detection was then applied to remove residual artifacts such as lead labels or patient identifiers. A morphological opening (erosion followed by dilation with a 3 × 3 kernel) further reduced edge noise and smoothed waveform boundaries. The binary image was inverted to display the waveform in white on a black background, followed by median blurring and histogram equalization to suppress noise and enhance contrast. Finally, the image was re-binarized to yield a clean, high-contrast ECG suitable for model input (Fig. 7).

### Spatial attention module

We used ConvNeXt-T as the feature extraction backbone, a compact and efficient CNN architecture incorporating transformer-inspired design elements. This variant offers a good trade-off between computational cost and accuracy, making it suitable for small medical datasets. To enhance spatial focus, we integrated lightweight spatial attention modules into ConvNeXt-T. These modules help the network emphasize salient regions while suppressing irrelevant background patterns—particularly valuable for ECG and respiration-curve images with subtle waveform details.

**Fig. 8:**
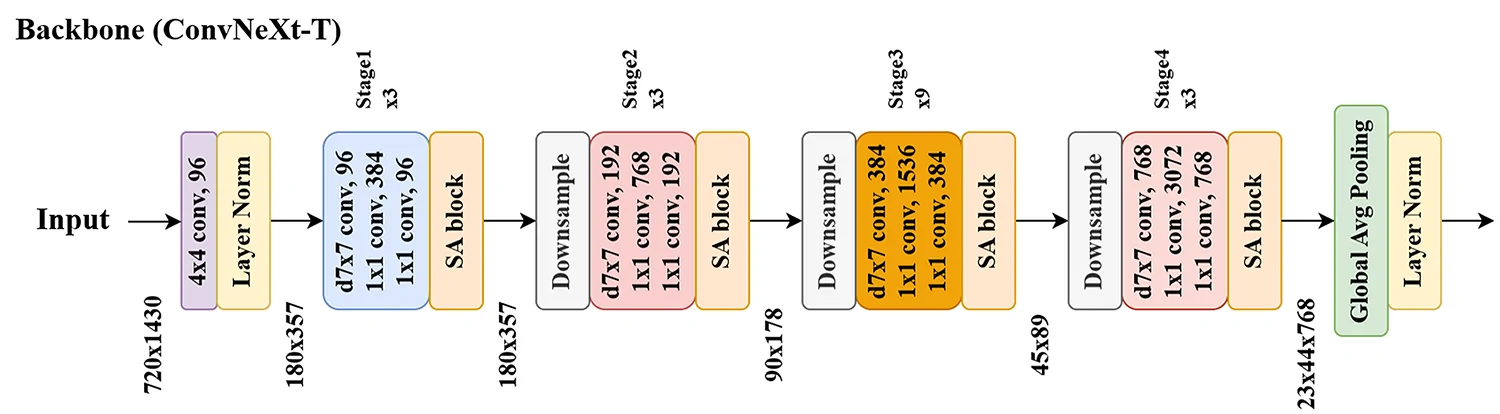
ConvNeXt-T with spatial attention blocks

Following the design of the Convolutional Block Attention Module (CBAM) [23], attention blocks were inserted after Stages 1–4 of ConvNeXt-T. This selective placement captures features across multiple spatial scales while avoiding overfitting in a limited dataset. The overall architecture with integrated spatial attention is shown in Fig. 8.

**Fig. 9:**
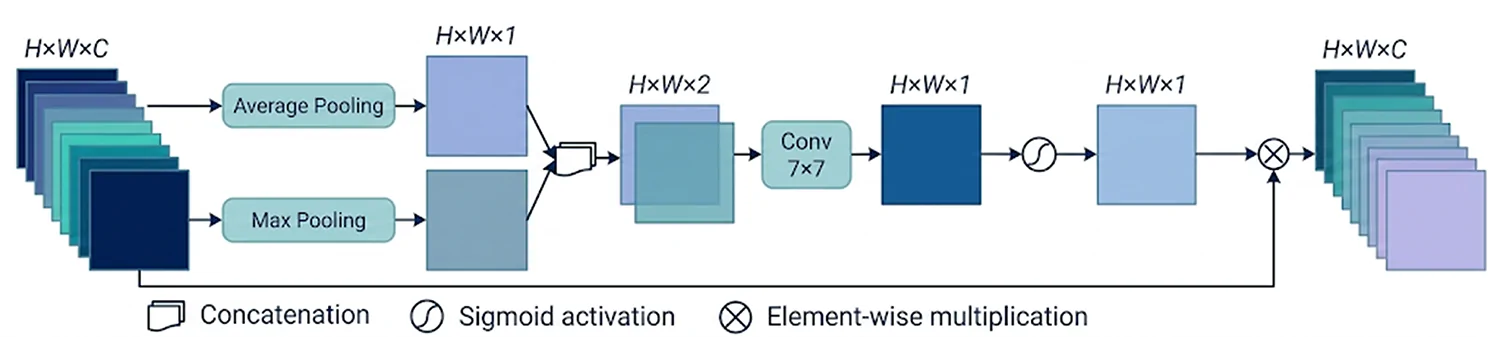
Spatial attention module

The spatial attention component operates on an intermediate feature tensor of size H ×W×C. First, we generated two 2D maps by performing average pooling and max pooling over the channel dimension, each producing a tensor of shape H × W × 1 [24]. We then stacked these two maps along the channel axis to form a H × W × 2 tensor, which was fed into a convolutional layer. After applying a sigmoid activation to the convolution output to generate the spatial attention mask, we multiplied this mask element‑wise with the original feature map to amplify critical spatial regions. The architecture of the spatial attention module is depicted in Fig. 9.

### Transfer learning for ECG branch

One major challenge is the limited ECG data in our NTUH dataset, which risks overfitting. To mitigate this, we pre-trained the ECG branch on an external ECG dataset from Far Eastern Memorial Hospital (FEMH) before fine-tuning on our internal data. The FEMH is also a tertiary academic medical center with approximately 1200 beds and 130,000 ED visits per year. The FEMH-ECG data set consisted of ECG images collected from ED patients presenting to the FEMH from 2016 to 2023. For the pretraining purposes, we randomly selected admitted cases and non-admitted controls from this ECG data set, resulting in 713 ECG reports (339 admitted, 374 non-admitted).

### Indicator-gated dual-branch fusion

**Fig. 10:**
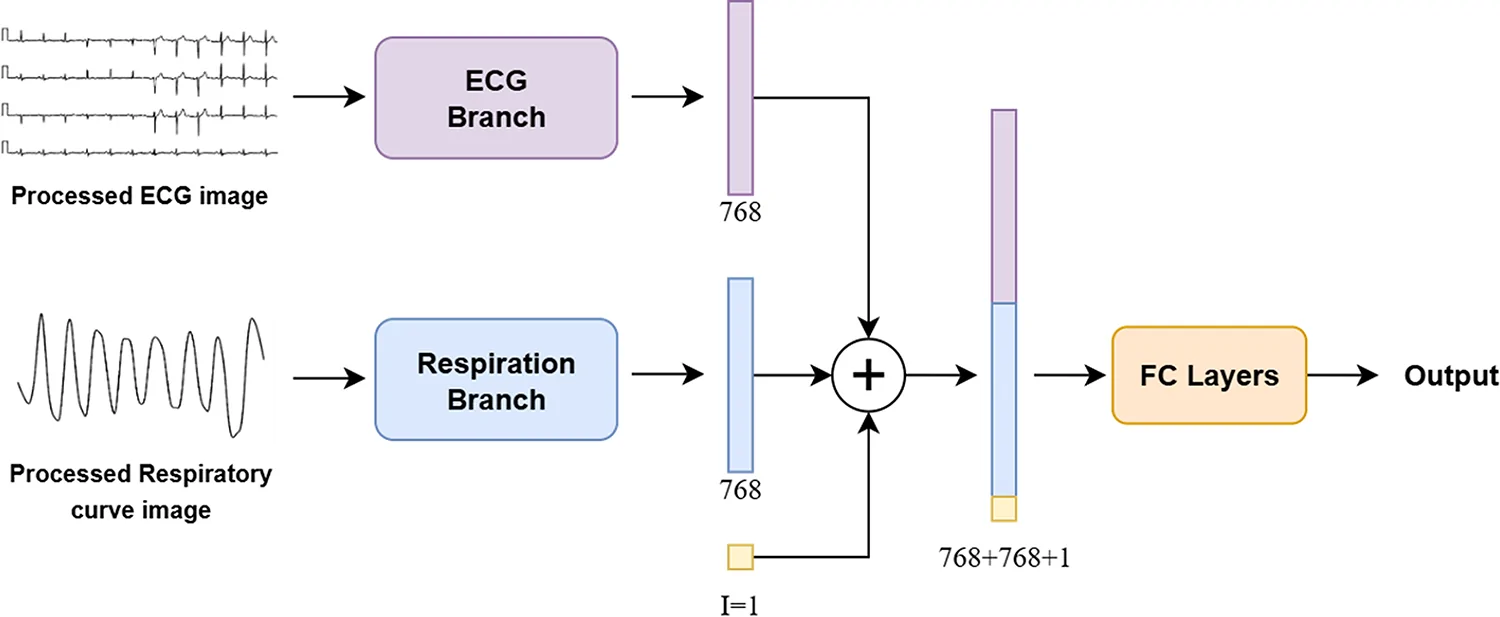
Fusion indicator. When I = 1, ECG and respiration features are merged with the indicator and passed to the full branch

**Fig. 11:**
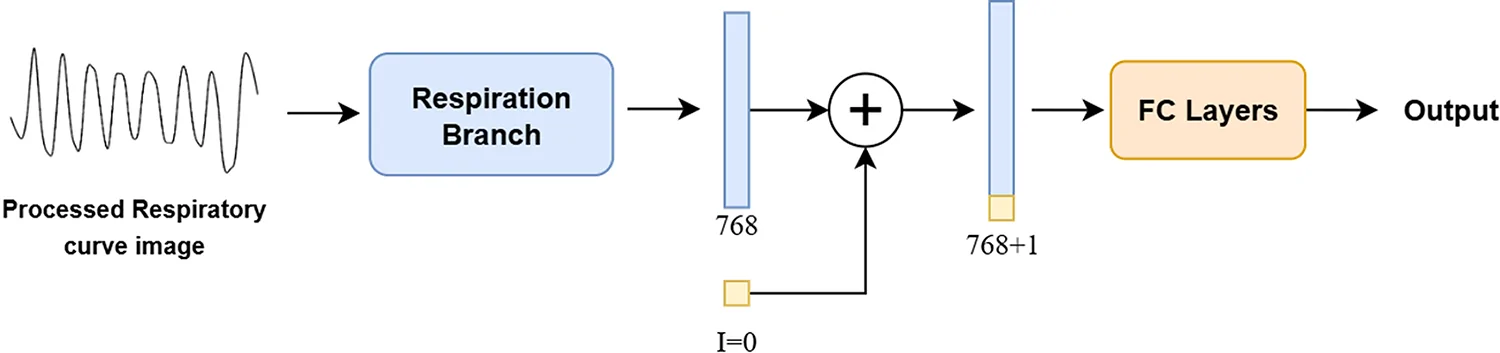
Fusion indicator. When I = 0, only respiration features and the indicator are used in the respiration-only branch

Because the performance of ECG at triage was affected by patients’ symptoms and triage nurses’ judgment, only a subset of patients from NTUH had ECG images available. To this end, we introduced a binary indicator I, defined as I = 1, ECG available, 0, ECG missing in the fusion stage. When I = 1, we concatenated the ECG feature vector e, the respiration feature vector r, and the indicator I into the input of the “full༂classifier branch (Fig. 10). When I = 0, we omitted e and instead concatenated r with I, feeding them into the “respiration-only༂branch (Fig. 11). This indicator-gated design enables the model to gracefully switch between the two branches, maintaining robust performance even when ECG data is missing.

### Augmented fusion with triage features (Baseline + Fusion)

To explore whether structured triage variables provided complementary information beyond the cardio-respiratory signals, we additionally evaluated an augmented configuration (‘Baseline + Fusion’) that combined the deep features used by the indicator-gated fusion model with the structured features used by the baseline logistic regression. This configuration uses feature-level (late) fusion. The deep ECG embedding e (when available), deep respiration embedding r, the binary ECG-availability indicator I, and the structured triage features (TTAS level, age, sex, heart rate, respiratory rate) were concatenated and passed through the same dual-branch indicator-gated MLP described above. The model was trained and evaluated using the same stratified 4-fold cross-validation splits and optimization settings as the indicator-gated fusion model.

### Class balanced loss

Our NTUH dataset exhibited a positive-to-negative sample ratio of approximately 1:2.7, causing standard losses to bias predictions toward the majority class. To counteract this, we employ the Class-Balanced Loss framework of Cui et al. [17], which reweights per-class contributions based on the “effective number” of samples. Given a class c with n_c_ samples and a hyperparameter β ∈ [0,1), the effective number Enc is defined as :

1 $$\:\begin{array}{c}{E}_{{n}_{c}}=\frac{1-{\beta\:}^{{n}_{c}}}{1-\beta\:}\end{array}$$

The corresponding Class-Balanced weight for class c is then given by

2 $$\:\begin{array}{c}{\alpha\:}_{c}=\frac{1}{{E}_{{n}_{c}}}=\frac{1-\beta\:}{1-{\beta\:}^{{n}_{c}}}\end{array}$$

Intuitively, classes with fewer samples (small n_c_) receive larger weights α_c_, while as β → 0 all α_c_ → 1, recovering the unweighted case. This framework can be applied on top of any base loss L(p, y) (e.g., cross-entropy, focal loss, Dice loss) by defining the Class Balanced loss (CB(p, y)) as :

3 $$\:\begin{array}{c}CB\left(p,\:y\right)=\:{\alpha\:}_{y}\cdot\:L\left(p,\:y\right),\end{array}$$

where p denotes model predictions, and y denotes the true label. For our binary classification task, we choose BinaryCross-EntropywithLogits (BCEWithLogits) as the base loss L(p, y), defined as:

4 $$\:\begin{array}{c}BCE\:With\:Logits\left(x,\:y\right)=y\cdot\:log\left(1+{e}^{-x}\right)+\left(1-y\right)\cdot\:\mathrm{log}\left(1+{e}^{x}\right),\end{array}$$

where x is the logit output by the model, and y ∈ {0,1} is the binary ground-truth label. This loss is particularly well-suited to binary classification tasks because it symmetrically penalizes false positives and false negatives through its two terms, ensuring balanced learning across both classes. In addition, BCEWithLogits operates directly on unnormalized logits x, internally applying the sigmoid activation for numerical stability, avoiding instability from computing log(0) or similar issues when probabilities are close to 0 or 1. By substituting L(p, y) = BCEWithLogits(x, y) into the Class-Balanced loss formulation, we obtain our final loss function Class-Balanced Binary Cross-Entropy with Logits (CB-BCEWithLogits), which is written explicitly as:

5 $$\:\begin{array}{c}CB-BCE\:With\:Logits\left(x,\:y\right)=\frac{1-\beta\:}{1-{\beta\:}^{{n}_{1}}}\cdot\:y\cdot\:\mathrm{log}\left(1+{e}^{-x}\right)+\frac{1-\beta\:}{1-{\beta\:}^{{n}_{0}}}\cdot\:(1-y)\cdot\:log(1+{e}^{x}),\end{array}$$

where n_1_ and n_0_ are the numbers of positive and negative samples, respectively. This formulation explicitly upweights the contribution of the minority (positive) class through the factor $\:\frac{1-\beta\:}{1-{\beta\:}^{{n}_{1}}}$, which is larger when n_1_ is small. As a result, the model is incentivized to reduce false negatives on the positive class, improving sensitivity, while still accounting for the majority class. This approach promotes equitable learning across classes and mitigates the bias toward the majority class inherent in standard loss functions.

### Experimental setup

**Fig. 12:**
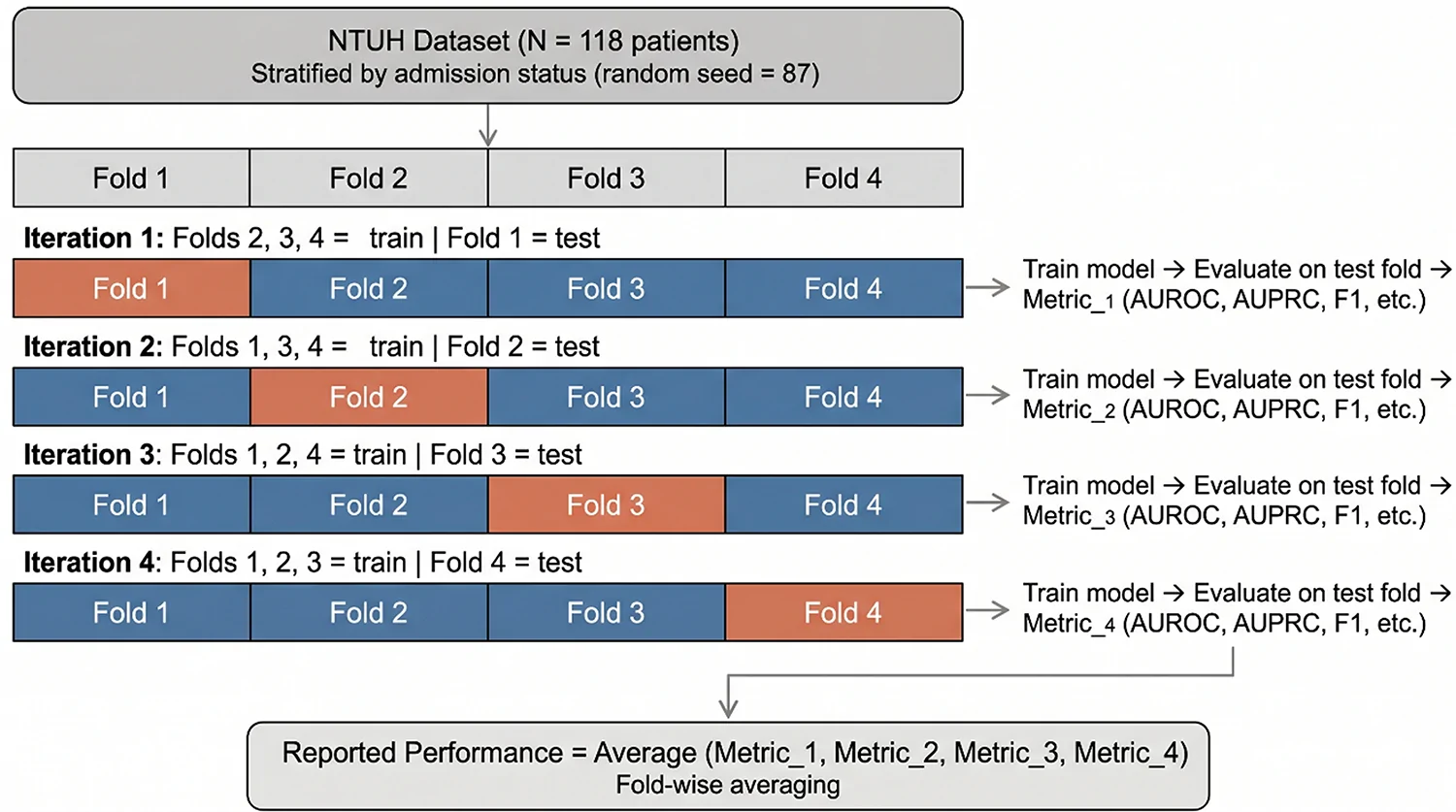
Evaluation workflow: stratified 4-fold cross-validation with fold-wise metric averaging

To comprehensively evaluate our approach, we assessed the performance of three different model configurations: the fusion model (Fusion), the ECG-only branch (ECG), and the respiration-only branch (Respiration). This design allows us to analyze the individual contributions of each modality and the effectiveness of our fusion strategy. Given the limited size of the NTUH dataset, we adopted a stratified 4-fold cross-validation scheme for both the Fusion and Respiration models. Although stratification was applied only to admission status, the resulting distribution of ECG availability across folds was approximately balanced (Fold 1: 9 ECG-available / 22 ECG-unavailable; Folds 2–4: 8 / 21 each). To avoid optimistic bias, we did not perform extensive hyperparameter tuning on the validation sets, obviating the need for nested cross-validation. Instead, hyperparameters were empirically fixed prior to evaluation based on established ConvNeXt best practices. Reported metrics were averaged across the four test folds (fold-wise averages); no separate held-out test set was used (random seed = 87). To prevent information leakage between training and test folds, our pipeline distinguishes three component categories: (a) *sample-independent fixed extractors* — the respiration signal pipeline (YOLOv5, the 3D-CNN+BiLSTM estimator, bandpass filter, and curve-image generation) operates on each patient independently and uses no NTUH dataset-level statistics; (b) *externally pretrained components* — the ECG branch is pretrained on the FEMH-ECG dataset and then fine-tuned per fold using only NTUH training data; (c) *per-fold trained components* — the fusion classifier and all data-derived normalization statistics are confined to the training folds and applied unchanged to the held-out test fold. The same 4-fold splits were used for the Baseline Logistic Regression and the Baseline + Fusion variant. The full evaluation workflow is shown in Fig. 12. For the ECG branch, we first pre-trained the model on the external FEMH ECG dataset using 5-fold cross-validation. After training, we evaluated the model’s generalization ability on the NTUH ECG subset, without further fine-tuning. This setup reflects the transferability of knowledge learned from a larger and more diverse dataset. To further validate the necessity of transfer learning, we also conduct a baseline experiment by training the ECG model directly on the NTUH ECG data using 3-fold cross-validation. The results were compared against the transfer learning setup to highlight the benefits of leveraging external data. All models were implemented in the PyTorch ecosystem [25] and were trained on an NVIDIA RTX 3090 GPU to leverage its high-throughput parallelism. Optimization uses the AdamW algorithm [26], which decouples weight decay from gradient updates to improve convergence. The model was trained for 150 epochs, with the initial learning rate set at 1 × 10 − 4 and a cosine-annealing learning-rate scheduler. Mixed-precision training was enabled with gradient accumulation over every five mini-batches to reduce memory usage and simulate a larger effective batch size, resulting in faster and more stable convergence.

### Evaluation metrics

In our binary classification task for hospital admission prediction using ECG images and respiratory videos, we employed several metrics derived from the confusion matrix, such as accuracy, precision, recall (sensitivity), and specificity. These metrics provided a detailed breakdown of how well the model distinguished between positive and negative cases, helping us understand not only the overall accuracy but also how well the model balanced false positives and false negatives. We also calculated the Area Under the Receiver Operating Characteristic Curve (AUROC), which measured the trade-off between true positive rates and false positive rates across different decision thresholds. Moreover, we incorporated the Area Under the Precision-Recall Curve (AUPRC) to evaluate performance, particularly in handling imbalanced datasets like ours, where the positive class is underrepresented. All reported metrics in cross-validation tables were computed per fold and then averaged across folds. To calculate the 95% confidence intervals for these metrics, 1,000 bootstrap iterations were performed using the percentile method. Model calibration was assessed by plotting predicted admission probabilities against actual admission probabilities across bins of predicted probabilities.

## Results

In this section, we first compared quantitatively against a recent study, then evaluated against the baseline model. Next, we conducted a series of ablation studies to quantify the impact of each core module. Finally, given the need for explainability in clinical AI, we used Score-CAM visualizations to illustrate the spatial regions driving our model’s decisions.

### Dataset

**Fig. 13:**
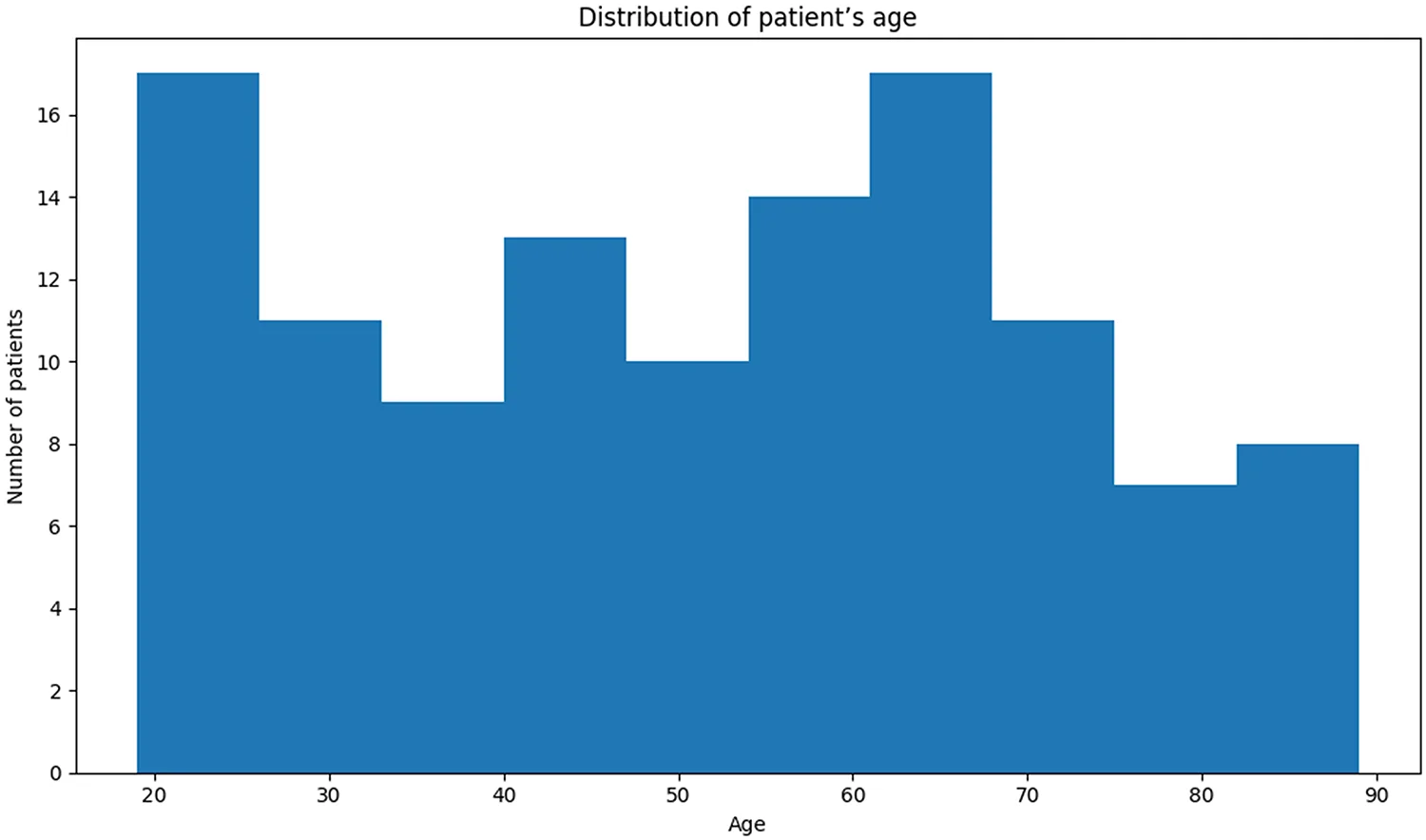
Distribution of patients’ age in the NTUH dataset

The NTUH dataset used in this study consists of clinical data collected from 118 patients in the ED of NTUH. It included both respiration videos and ECG images if available. Figure 13 shows the distribution of patient ages in the NTUH dataset, which spans a broad adult age range. Additionally, Table 1 summarizes patient demographics and clinical characteristics, including gender ratios, Taiwan Triage and Acuity Scale (TTAS) levels, and hospital admission rates.

**Table 1:** Patient characteristics in the NTUH dataset

Variable	Enrolled ED Patients [*n* (%)]	Admission [*n* (%)]
Total	118 (100)	32 (27.12)
Male	55 (46.61)	22 (40)
Female	63 (53.69)	10 (15.87)
**Triage TTAS score**		
1 (highest acuity)	0 (0)	0 (0)
2	6 (5.08)	3 (50)
3	103 (87.29)	29 (28.16)
4	7 (5.93)	0 (0)
5 (lowest acuity)	2 (1.69)	0 (0)
ECG availability	33 (27.9)	18 (54.5)

### Experimental results

#### External comparison

Table 2 compares our study with Ip et al. [14]. Their dataset comprised 723 ED patients, with an admission rate of 40.9%. They trained three separate Random Forest classifiers, one on short triage video features, one on structured clinical data, and one on chief-complaint text. They then combined the outputs of these classifiers via late fusion to predict hospital admission. They also reported Negative Predictive Value (NPV), which measures the proportion of true negatives among all cases predicted negative. A high NPV indicates the model can reliably rule out low-risk patients, reducing the chance of discharging individuals who actually require admission. Although the reported metrics in our cohort numerically exceed those of Ip et al., direct comparison is limited by differences in cohort characteristics, admission rates, input modalities, and modeling approaches; the comparison is therefore offered as descriptive context rather than as a head-to-head benchmark.

**Table 2:** Comparison of our work with [14]

Method	Recall	Precision	Specificity	NPV	AUROC	AUPRC
Ip et al.	0.665	0.558	0.626	0.738	0.714	0.642
Ours	0.749	0.635	0.838	0.798	0.863	0.675

Abbreviations: AUROC = area under the receiver operating characteristic curve; NPV = negative predictive value

#### Compare to benchmark

Prior work in hospital admission prediction often benchmarks against a reference model based on logistic regression using the Emergency Severity Index (ESI). In our context, we adopted a similar logistic regression baseline using the Taiwan Triage and Acuity Scale (TTAS), which is widely used in Taiwan. The baseline was a standard logistic regression with L2 regularization, specified as P(admission = 1) = σ(β₀ + β₁ × TTAS + β₂ × age + β₃ × sex + β₄ × heart_rate + β₅ × respiratory_rate), where TTAS was coded as an integer ordinal variable (range 1–5), and the remaining variables were used in their native scales. None of the included variables had missing values in the NTUH dataset, so no imputation was required. The baseline was trained and evaluated using the same stratified 4-fold cross-validation splits described in the Experimental Setup. Table 3 summarizes the performance of our three models—ECG branch, respiration video branch, and full fusion model—against the enhanced logistic regression baseline. Due to the limited ECG sample size, the ECG-only model showed high AUPRC (driven by a relatively high positive rate) but performed poorly on all other metrics. The respiration-only model, using just video data, outperformed the baseline model on every metric except AUPRC, achieving an AUROC of 0.810. By integrating ECG and respiration via our indicator-gated fusion, the fusion model further improved all metrics over the respiration branch except recall, most notably increasing AUROC to 0.863. Finally, an augmented configuration combining the indicator-gated fusion model with the structured triage features used by the baseline (Baseline + Fusion; see Methods, Augmented Fusion with Triage Features) achieved a further AUROC gain to 0.893, indicating that structured triage variables and cardio-respiratory deep features may carry complementary information.

**Table 3:** Comparison of the model performance of a baseline model and various components of our proposed models

Method	Acc	Recall	Precision	F1-score	AUROC	AUPRC
Baseline model*	0.644 (0.559–0.729)	0.625 (0.441–0.800)	0.400 (0.255–0.538)	0.488 (0.333–0.615)	0.730 (0.619–0.832)	0.522 (0.333–0.691)
ECG-only (*N* = 33)	0.697 (0.545–0.848)	0.556 (0.316–0.778)	0.833 (0.600–1.000)	0.667 (0.435–0.839)	0.693 (0.488–0.863)	0.724 (0.483–0.922)
Respiration-only	0.788 (0.712–0.864)	0.750 (0.588–0.899)	0.588 (0.444–0.747)	0.656 (0.515–0.768)	0.810 (0.714–0.899)	0.514 (0.385–0.718)
ECG + Respiration	0.814 (0.737–0.881)	0.749 (0.584–0.887)	0.635 (0.469–0.796)	0.680 (0.518–0.794)	0.863 (0.788–0.934)	0.675 (0.507–0.844)
Baseline + Fusion	0.839 (0.771–0.898)	0.915 (0.817-1.000)	0.647 (0.509–0.792)	0.754 (0.625–0.848)	0.893 (0.826–0.946)	0.729 (0.571–0.870)

The numbers in parentheses are 95% confidence intervals

Note: All models were evaluated using the same stratified 4-fold cross-validation splits at the patient level. The ECG-only row reflects performance on the *N* = 33 ECG-available subset and is therefore not directly comparable to the other rows, which were evaluated on the full *N* = 118 cohort

Abbreviations: Acc = Accuracy; AUROC = area under the receiver operating characteristic curve; AUPRC = area under the precision-recall curve. * Includes Taiwan Triage and Acuity Scale, age, sex, heart rate, and respiratory rate

To assess stability across fold partitions and random initialization, we repeated the full cross-validation of the ECG+Respiration model under three additional predefined random seeds; across all four seeds (87, 42, 123, 2026), the model achieved an accuracy of 0.755–0.814, a recall of 0.749–0.771, a precision of 0.554–0.635, an F1-score of 0.616–0.680, an AUROC of 0.843–0.863, and an AUPRC of 0.590–0.675. Performance remained similar across seeds, indicating that the reported results did not rest on a single favorable split.

### ECG model performance

We first evaluated the ECG branch on the larger FEMH-ECG dataset using 5-fold cross-validation to establish its baseline performance, as shown in Table 4. Next, we tested the pretrained ECG branch directly on the NTUH ECG subset without any fine-tuning to assess transferability, and compared this against an in-domain model trained solely on NTUH ECG data via 3-fold cross-validation. Table 5 shows that pre-training yields a substantial AUROC gain (0.693 vs. 0.578). Due to the limited size of the NTUH ECG subset, metrics other than AUROC were not reliable.

**Table 4:** Performance on FEMH-ECG Dataset

Method	Acc	Recall	Precision	F1-score	AUROC	AUPRC
FEMH-ECG	0.678	0.762	0.634	0.692	0.740	0.675

Abbreviations: Acc = Accuracy; AUROC = area under the receiver operating characteristic curve; AUPRC = area under the precision-recall curve

**Table 5:** Transferability to NTUH ECG Subset

Method	Acc	Recall	Precision	F1-score	AUROC	AUPRC
No Pretraining	0.606	0.833	0.604	0.684	0.578	0.615
Pretrained	0.697	0.556	0.833	0.667	0.693	0.724

Abbreviations: Acc = Accuracy; AUROC = area under the receiver operating characteristic curve; AUPRC = area under the precision-recall curve

### Subgroup analysis of ECG availability

To evaluate the benefit of electrocardiogram (ECG) data among patients with both ECG and respiration videos available, we conducted a subgroup analysis limited to the 33 patients with both modalities (Table 6). Within this cohort, integrating ECG data with respiratory videos (the Fusion model) improved the model F1-score, AUROC, and AUPRC slightly. Notably, the recall increased from 0.611 to 0.944, suggesting this subgroup had a relatively higher actual admission rate than the overall group that the Fusion model learned from.

**Table 6:** Subgroup analysis of model performance on patients with available ECG data (*N* = 33)

Method	Acc	Recall	Precision	F1-score	AUROC	AUPRC
ECG-only	0.697 (0.545–0.848)	0.556 (0.312–0.790)	0.833 (0.600–1.000)	0.667 (0.429–0.833)	0.693 (0.482–0.857)	0.724 (0.484–0.917)
Respiration-only	0.606 (0.455–0.758)	0.611 (0.375–0.824)	0.647 (0.400–0.867)	0.629 (0.400–0.783)	0.585 (0.371–0.805)	0.547 (0.355–0.791)
ECG + Respiration	0.576 (0.394–0.727)	0.944 (0.809–1.000)	0.567 (0.379–0.733)	0.708 (0.524–0.830)	0.600 (0.393–0.790)	0.610 (0.391–0.860)

Abbreviations: Acc = Accuracy; AUROC = area under the receiver operating characteristic curve; AUPRC = area under the precision-recall curve

### Model calibration curve

**Fig. 14:**
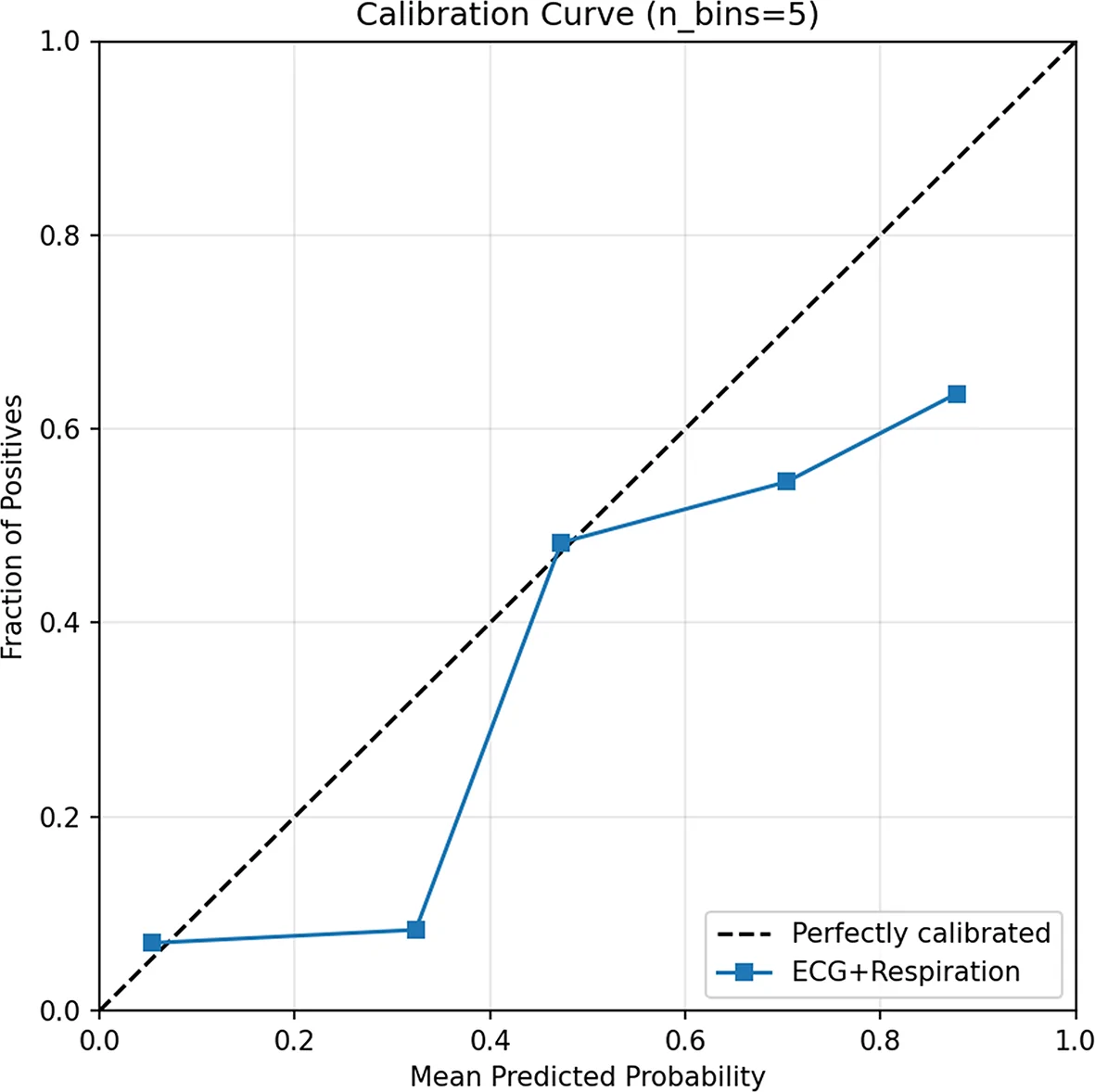
Calibration curve for the ECG-respiration fusion model

We evaluated the calibration of the full fusion model using pooled out-of-fold predictions from the stratified 4-fold cross-validation (*N* = 118), ensuring each prediction was generated by a model that had not seen that patient during training. The calibration curve was constructed using 5 bins (Fig. 14); a 5-bin configuration was selected to ensure adequate sample sizes within each bin, given the overall dataset size. The calibration curve indicated that the model was generally well-calibrated, with a gradual increase in admission probability across bins, although some degree of overestimation of actual probabilities of admission was noted. To complement the visual assessment, we report two quantitative calibration measures: a Brier score of 0.161 (computed as the mean squared error between predicted probabilities and observed binary outcomes; for reference, the no-information Brier score at the cohort admission prevalence of 27.1% is 0.198, so the model’s value reflects a meaningful improvement over the prevalence-only baseline) and an Expected Calibration Error (ECE) of 0.094 (computed by partitioning predictions into 5 equal-width bins and taking the sample-size-weighted mean absolute difference between predicted probability and observed event frequency).

### Ablation study

Table 7 presents an ablation study on the respiration-only branch, examining the effects of Spatial Attention (SA) and Class-Balanced Loss (CBL). Both modules independently boosted performance: SA sharpened feature extraction by focusing on key regions, enhancing AUROC, recall, and precision, while CBL directly tackled class imbalance, driving larger gains in overall accuracy and precision. When combined, SA and CBL complemented each other to deliver the greatest improvements across all metrics except precision, confirming the value of attention-guided feature weighting and balanced learning.

**Table 7:** Ablation study for the Respiration model

Modules	Acc	Recall	Precision	F1-score	AUROC	AUPRC
None	0.721	0.687	0.507	0.561	0.768	0.501
SA only	0.736	0.719	0.595	0.609	0.803	0.503
CBL only	0.779	0.718	0.619	0.634	0.791	0.483
SA + CBL	0.788	0.750	0.588	0.656	0.810	0.514

Abbreviations: Acc = Accuracy; AUROC = area under the receiver operating characteristic curve; AUPRC = area under the precision-recall curve; SA = spatial attention; CBL = class balanced loss

Table 8 presents an ablation study on our full fusion model, isolating the effects of the Spatial Attention (SA) module and Class-Balanced Loss (CBL). Overall, the trends mirrored those observed in the respiration-only branch but with higher baseline performance. Incorporating SA improved AUROC, recall, and F1-score by guiding the network to key cardiopulmonary features; however, it caused a slight drop in precision as attention shifts may introduce minor false positives. Applying CBL alone yielded substantial gains in accuracy and recall but noticeably reduced precision, reflecting a trade-off inherent to balancing class frequencies. When both SA and CBL were combined, the model achieved the highest overall metrics.

**Table 8:** Ablation study for the fusion model

Modules	Acc	Recall	Precision	F1-score	AUROC	AUPRC
None	0.756	0.637	0.648	0.577	0.819	0.636
SA only	0.797	0.621	0.646	0.617	0.844	0.621
CBL only	0.787	0.688	0.607	0.635	0.845	0.656
SA + CBL	0.814	0.749	0.635	0.680	0.863	0.675

Abbreviations: Acc = Accuracy; AUROC = area under the receiver operating characteristic curve; AUPRC = area under the precision-recall curve; SA = spatial attention; CBL = class balanced loss

Table 9 evaluates two key design choices, ECG pretraining and the binary indicator, on our full Fusion model with SA and CBL enabled. Omitting ECG pretraining led to a drop in AUROC but a slight increase in recall, demonstrating that transfer learning from external ECG data improves overall discrimination. Removing the binary indicator, which simply zeroed out missing ECG inputs rather than gating them, causes a notable decrease in recall with a small gain in precision, reflecting overreliance on respiratory features when ECG was absent. In contrast, the full model, which used both pretrained ECG weights and explicit modality signaling, achieved the best balance across all metrics, underscoring the importance of these strategies for robust multimodal fusion.

**Table 9:** Ablation of ECG Pretraining and Modality Indicator in the Fusion Model

Method	Acc	Recall	Precision	F1-score	AUROC	AUPRC
w/o ECG Pretraining	0.728	0.764	0.542	0.608	0.806	0.552
w/o Indicator	0.709	0.692	0.644	0.529	0.835	0.614
Full fusion model	0.814	0.749	0.635	0.680	0.863	0.675

Abbreviations: Acc = Accuracy; AUROC = area under the receiver operating characteristic curve; AUPRC = area under the precision-recall curve

### Visualization of model attention on respiratory curve

**Fig. 15:**
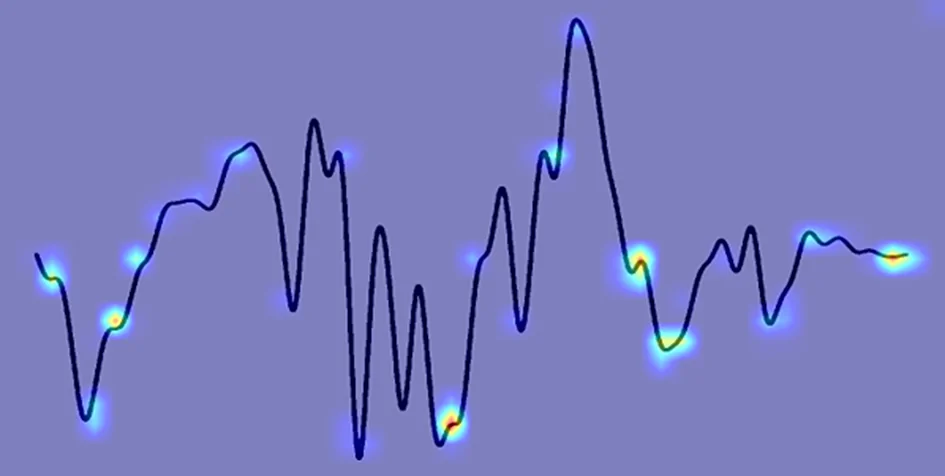
Score-CAM visualization for a positive respiratory curve sample

**Fig. 16:**
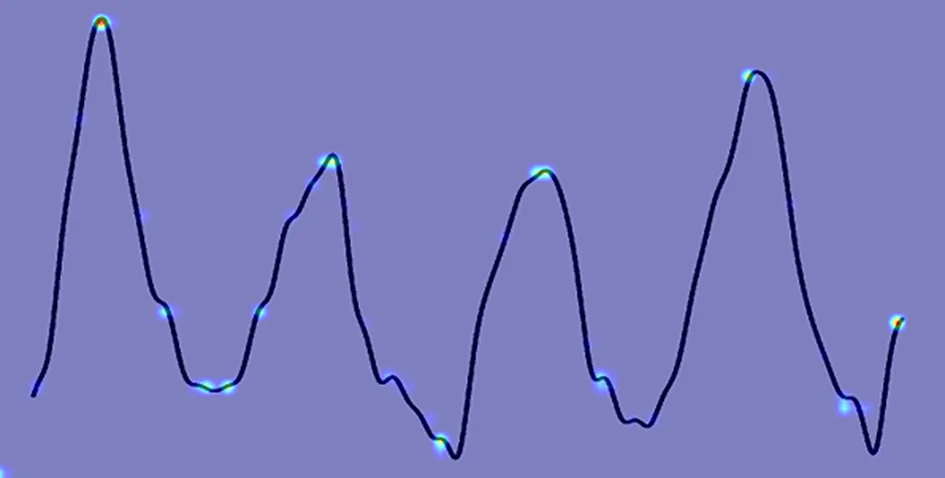
Score-CAM visualization for a negative respiratory curve sample

We applied Score-CAM to visualize the regions of the respiratory curve image that our fusion model attends to when predicting hospital admission. Figures 15 and 16 show representative overlays for a positive (admitted) and a negative (non-admitted) case, respectively. In both examples, the model consistently highlighted the inflection points of the respiratory waveform, which corresponded to peaks and troughs in the curve. These inflections are physiologically meaningful: the vertical excursion between peak and trough reflects respiratory amplitude (chest wall movement and effort), while the temporal spacing of consecutive inflections indicates breathing frequency (rate and severity of dyspnea). By focusing on these features, our model leverages key indicators of respiratory pattern to inform its admission predictions.

For example, the respiration curve in Fig. 15 is from a 62-year-old female patient with multiple comorbid conditions, including anemia and end-stage renal disease, presenting to the ED with severe abdominal pain (7 out of a pain scale from 0 to 10). The respiration curve showed irregular, rapid respirations, indicating severe respiratory distress, likely due to abdominal pain and anemia. She was later diagnosed with acute pancreatitis and admitted to the hospital. The respiration curve in Fig. 16 is from a 33-year-old man with no underlying diseases who also presented to the ED with mild abdominal pain (4 out of 10) and diarrhea. The smooth respiration showed no respiratory distress. He was diagnosed with acute gastroenteritis and was sent home soon. The two examples underscored that respiratory distress may contain clues to complex, underlying ongoing disease processes. Of note, Score-CAM visualizations are hypothesis-generating and do not represent causal relationships.

### Independent prospective holdout cohort

To assess the temporal stability of the proposed model at the same site, we evaluated it on an independent prospective holdout cohort (*N* = 20) enrolled after the primary study period. These data were entirely unseen by the model during training. As shown in Table 10, the fusion model maintained comparable performance, with an AUROC of 0.780 (95% CI: 0.562–0.970), an AUPRC of 0.811 (95% CI: 0.514–0.974), and an accuracy of 0.750 (95% CI: 0.550–0.950). Because the cohort is small and was enrolled at the same site, these results suggest temporal stability rather than broader generalizability; multi-site validation remains a necessary next step.

**Table 10:** Performance on the independent prospective holdout cohort (*N* = 20)

Method	Acc	Recall	Precision	F1-score	AUROC	AUPRC
ECG + Respiration	0.750 (0.550–0.950)	0.800 (0.545-1.000)	0.727 (0.429-1.000)	0.762 (0.500-0.933)	0.780 (0.562–0.970)	0.811 (0.514–0.974)

Abbreviations: Acc = Accuracy; AUROC = area under the receiver operating characteristic curve; AUPRC = area under the precision-recall curve. Values in parentheses are 95% bootstrapped confidence intervals

## Discussion

In this study, we developed a multimodal deep learning-based framework that leveraged respiratory video and ECG images to predict hospital admission at ED triage. The main fusion model achieved excellent discrimination (AUROC = 0.863) while offering interpretable attention maps on respiratory waveforms. An exploratory augmented configuration that additionally incorporated basic triage features (age, sex, TTAS score, heart rate, and respiratory rate) yielded a higher AUROC of 0.893, suggesting that combining structured triage variables with cardio-respiratory deep features may further improve discrimination. These results suggest that cardio-respiratory signals and videos can be combined with traditional triage structural data as a multimodal ED triage system in the future.

Our previous deep learning-based studies on ED disposition prediction have predominantly relied on structured clinical data and/or free-text chief complaints [27, 28]. With the advancement in computer vision, recent work has begun to explore short triage videos as a novel input modality and has shown encouraging results [14]. The major difference between our study and Ip et al. is that we specifically extracted cardiac and respiratory signals from images for admission prediction at triage, while their videos captured patients’ general appearance and behaviors during the ED stay. While their approach may contain more information, it may be difficult for model training purposes without specific labeling or noise filtering. Although our model performance seemed better than theirs, direct comparisons are difficult due to differences in cohort characteristics, admission practices, and feature sets. Nonetheless, the bottom line would be that video-based features are informative and seem to capture some aspects of clinicians’ “clinical gestalt,” yet classical triage variables remain complementary and useful predictors. The observed AUROC gain in the exploratory Baseline + Fusion configuration suggests that systems integrating both automated visual assessment and succinct clinical measurements may be valuable in future triage practice, though this finding warrants confirmation in independent data.

Our model has several technical innovations. For the first time, we presented a multimodal deep-learning framework that predicts hospital admission at ED triage by jointly leveraging respiratory videos and 12-lead ECG images. In addition, our pipeline automatically extracts and denoises respiratory waveforms from unconstrained triage videos and preprocesses ECG images before feature extraction with ConvNeXt-T enhanced by spatial attention. This could potentially facilitate real-time deployment in the ED setting. Third, an indicator-gated fusion mechanism, transfer-learning for the ECG branch, and a class-balanced BCEWithLogits loss address modality missingness and class imbalance, all of which are frequently encountered in the real world. Of note, because ECG availability itself was associated with admission (as shown in Table 1), the ECG modality may serve as a proxy for clinician triage behavior in addition to physiological signals. Moreover, the fusion model outperformed single-modality variants, and ablation studies confirm the value of attention, transfer learning, and balanced loss. Finally, score-CAM visualizations further show the model attends to clinically meaningful respiratory features, improving model interpretability.

While our multimodal model seems promising and patient data were prospectively collected, the model was evaluated offline rather than in a real-time clinical workflow. Consequently, the logical next steps should include prospective validation in live ED settings to evaluate real-world performance, calibration, and the extent to which the model can provide actionable lead time for clinical intervention. Future research directions may also include expanding external validation across institutions and patient populations to test the model’s generalizability. In addition, it may be sensible to extract other types of information from images/videos (e.g., heart rate, blood pressure, distress, and pain) for model fusion purposes, as we successfully developed a deep learning-based model for pain estimation via facial videos [29]. Finally, conducting human-AI comparative studies is warranted to quantify whether and how model outputs improve usual care and patient outcomes.

This study has several potential limitations. First, the NTUH dataset size is modest, and the NTUH ECG subset is particularly small. Although we mitigated the risk of overfitting by transfer learning on an external ECG dataset, lightweight model architecture, cross-validation, and weight decay regularization, further validation on larger and more diverse cohorts is required. Second, as with all studies on ED admission, the admission decisions can vary across clinicians (e.g., different thresholds for admission) and institutions (e.g., ED crowding and bed availability) [30] and are influenced by social factors (e.g., family and social support), which adds additional variability in this outcome label. Third, modality missingness (e.g., patients without ECG images) remains a practical challenge. Although our subgroup analysis indicated that incorporating ECG data slightly improves model performance, the small sample size limits the model’s statistical reliability. Our findings regarding multimodal fusion should therefore be interpreted as preliminary, and future studies should consider extracting ECG directly from images if possible. Fourth, the triage-augmented experiment used a limited set of basic features; richer EHR information (past medical history, medication, prior hospitalizations) and other modalities (audio) might further improve performance but raise data-access and deployment complexities. Fifth, patients requiring immediate resuscitation and those who were on isolation precautions were excluded, thereby limiting the model’s generalizability and resulting in possible spectrum bias. A completely non-contact, ambient AI system would be needed to capture these important ED populations. Finally, ethical, privacy, and logistical considerations around routine video capture in clinical areas will need to be addressed before clinical deployment.

## Conclusions

In summary, our study demonstrates that visual signals extracted from triage videos and ECG images contain clinically meaningful information for predicting hospital admission, and that the addition of a small set of basic triage features substantially improves discrimination. The combination of interpretable attention visualizations and targeted modeling strategies (transfer learning, indicator gating, class-balanced loss) yields a promising framework that could inform future ED triage. Given the single-center nature of this study, further prospective, multi-site evaluations are warranted to validate generalizability and address operational and ethical considerations before clinical adoption.

## Supplementary Information

Below is the link to the electronic supplementary material.

Supplementary Material 1

## Data Availability

The artificial intelligence algorithm cannot be made publicly available because it is proprietary intellectual property. The computer code and data for this study are available from the corresponding authors upon reasonable request for research studies. To facilitate reproducibility, the model architecture, configuration, training hyperparameters, random seeds, and software package versions are detailed in Supplementary Material, and the preprocessing steps are described in the Methods.

## Abbreviations

Acc: Accuracy
AUPRC: Area under the precision-recall curve
AUROC: Area under the receiver operating characteristic curve
CBLoss: Class Balanced Loss
ECG: Electrocardiography
ED: Emergency Department
FEMH: Far Eastern Memorial Hospital
NTUH: National Taiwan University Hospital
SA: Spatial Attention
Score-CAM: Score-Class Activation Mapping
TTAS: Taiwan Triage Acuity Score
