# Supplementary material for: Cardio-respiratory deep learning model to predict hospital admission from emergency department electrocardiogram images and respiration videos: a prospective study
Source: BioData Min. 2026 Jul 8;19:77. doi: 10.1186/s13040-026-00583-9 (PMC13563894; doi:10.1186/s13040-026-00583-9)
Supplement: Supplementary file 1 [file 13040_2026_583_MOESM1_ESM.pdf]

Online Supplemental Material.

Figure. The participant flow chart.

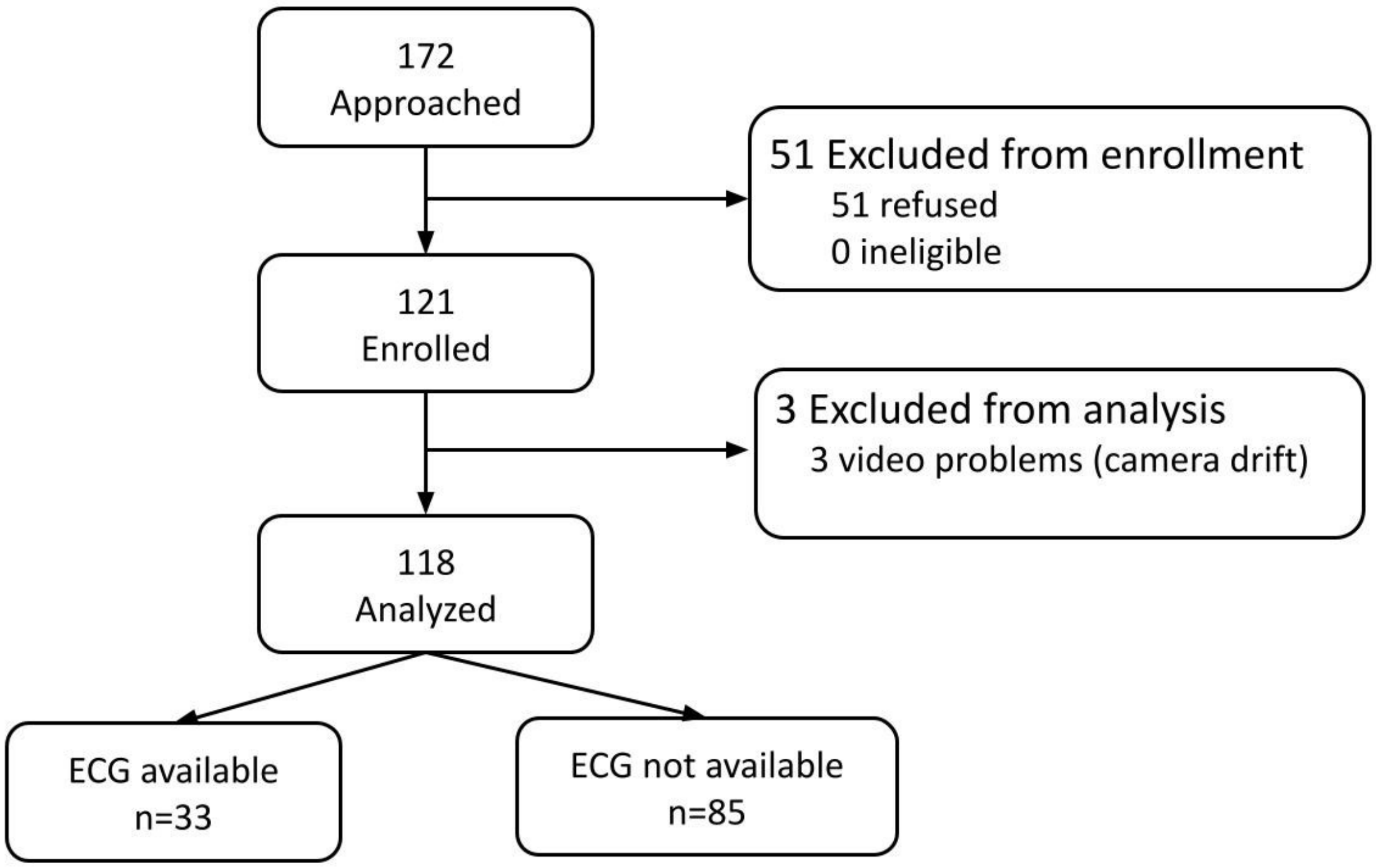

Supplementary Note. Implementation details for reproducibility.

This note provides component-level specifications complementing the Methods; settings already described in the main text are not repeated.

Model architecture. Each ConvNeXt-T backbone uses four stages with [3, 3, 9, 3] blocks and dimensions [96, 192, 384, 768]; a 4×4 stride-4 patchify stem; 2×2 stride-2 downsampling between stages; and per block, depthwise 7×7 convolution → LayerNorm → expansion-4 linear → GELU → linear → DropPath, yielding a 768-d vector after global average pooling. Each spatial-attention module computes channel-wise mean and max maps → 7×7 convolution → sigmoid, applied multiplicatively, after each stage.

Indicator-gated fusion head. Full branch ($I = 1$): Linear(1537→512) → ReLU → Dropout(0.3) → Linear(512→256) → ReLU → Dropout(0.2) → Linear(256→1), with the 1537-d input formed from the ECG (768-d), respiration (768-d), and indicator. Respiration-only branch ($I = 0$): identical structure with a 769-d input.

Training (additional settings). Batch size 12; weight decay 1×10^{-2}; class-balanced BCE-with-logits loss with $\beta = 0.9999$. Inputs are converted from grayscale to three channels by replication, with no further normalization or augmentation.

Random seeds. Primary results use a fixed seed of 87 (governing fold partitioning and initialization); stability was additionally assessed with predefined seeds 42, 123, and 2026.

Software environment. Python 3.8, PyTorch 1.13.1, torchvision 0.14.1, timm 0.6.12, CUDA 11.6, NumPy 1.21.6, SciPy 1.7.3, scikit-learn 1.1.0, OpenCV 4.6.0, Pillow 9.2.0, YOLOv5 (commit 485da42)
